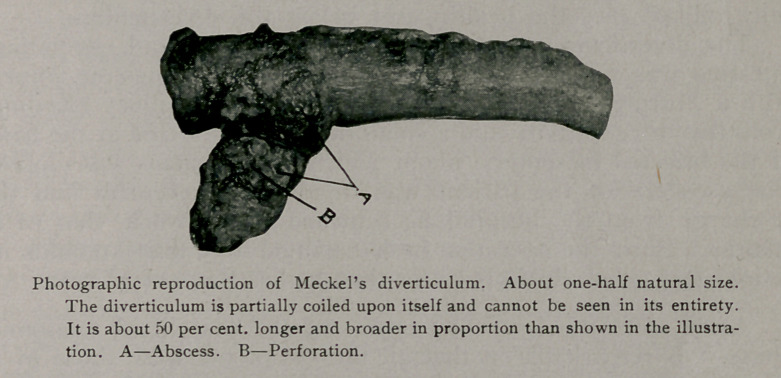# Intestinal Surgery

**Published:** 1904-06

**Authors:** Marshall Clinton

**Affiliations:** Buffalo, N. Y., Attending Surgeon, Sisters of Charity, and Erie County Hospitals, Buffalo, N. Y.; Instructor in Surgery in the University of Buffalo


### Intestinal Surgery.

### A Case of Acute Intestinal Obstruction due to Perforation of an Inflamed Meckel's Diverticulum.

By MARSHALL CLINTON, M. D., Buffalo, N. Y.,

Attending Surgeon, Sisters of Charity, and Erie County Hospitals, Buffalo, N. Y.; Instructor in Surgery in the University of Buffalo.

IN itself Meckel's diverticulum is of sufficient rarity to attract the interest of the physician; when it becomes diseased it demands attention. We are frank to admit the difficulty in diagnosticating the beginning point of many septic lesions in the abdomen before operating, just as it is difficult to determine the nature of the many masses felt in the belly before it is opened.

In cases of serious obstructive lesions of the intestine the safety of the patient depends on prompt operative intervention; such procedure being adopted with a clear view as to the condition, a full realisation of its gravity and a well-defined idea as to methods to be adopted for its relief. Intestinal surgery means prompt and fearless intervention; delay in the majority of cases means death. The lesion undoubtedly would be discovered and removed but the patient would die, thus placing another case among the archives of the professional humorist for reference as an instance wherein "the operation was successful, but the patient died."

The case reported herewith is interesting in itself as an anatomic rarity; it is doubly interesting as illustrating a rare and unusual condition wherein prompt operation before serious damage had been done, was followed by complete recovery.

The patient, a man of 44, was admitted to the Sisters of Charity Hospital August 8, 1903, in charge of Dr. Borzilleri. He had been suffering with colicky pains in the abdomen for five days and had been constipated. For 48 hours prior to his admission to the hospital there had been no movement whatever. Thirty-six hours before his admission he began to vomit material made up of bile-stained mucus and stomach contents. His temperature was 100° F.; pulse, 112; respirations, 22, costal in type. His tongue was dry, the blood pressure was low and a leukocytosis (23,000) was present. The patient was restless and anx-

Photographic reproduction of Meckel's diverticulum. About one-half natural size. The diverticulum is partially coiled upon itself and cannot be seen in its entirety. It is about 50 per cent. longer and broader in proportion than shown in the illustration. A—Abscess. B—Perforation.

ious and complained of a constant pain in the abdomen. He could not locate the pain definitely, but when asked to place his hand where it was most intense, passed both hands with a sweeping motion all over the abdominal region, saying: "It's here." Examination showed a rigid abdomen, moderately distended. On gentle pressure the patient bitterly complained of pain in all regions of the abdomen, and especially was this so over the lower quadrants of both sides.

From the history and examination a diagnosis was made of obstruction due probably to a perforative appendicitis with an advancing peritonitis. The patient was prepared for operation, the anesthetic used being ether. The operation was performed 17 hours after his admission to the hospital. The usual incision was made over the head of the cecum, which was easily reached and delivered into the operation wound. It was normal in condition as was the apendix, there being not the slightest sign even of irritation. Intraäbdominal digital examination detected the omentum adherent to a mass on the side near the middle line in the upper left quadrant quite as large as a closed fist, but without any determinable characteristics of a marked type.

The appendical wound was rapidly closed and an opening made in the middle line 3 inches in length. A few coils of distended small intestine floated into the wound. Following the course of this gut downward the previously discovered mass was disclosed. It was covered with omentum. The outer wall was formed by a coil of ileum about 8 inches long, which at each end was sharply flexed on its axis and densely adherent to the base of its mesentery. It was dark and edematous, but not gangrenous. Gentle separation of the adhesions with the fingers was followed by a thick flow of pus from a small abscess under the omentum and between the two sides of the coil of small intestine. The abscess cavity was cleansed and in it lay a Meckel's diverticulum 2½ inches long and 1⅜ inches in diameter, coiled on itself and with a perforation in its distal extremity. This diverticulum was found to spring from the ileum several feet away from the obstructed coil. The contents of the distended intestine were carefully milked into the healthy gut below the diverticulum.

The diverticulum with the adjacent inflamed and edematous intestine was resected and the free ends of the intestine joined with a Murphy button. There was persistent capillary bleeding from the abscess cavity and a small drain was inserted at the base of the infected mesentery, about which the abdomen was closed. The recovery of the patient was rapid and uneventful and on discharge from the hospital he returned to his work, that of a laborer. Since the operation he has gained over thirty pounds in weight and generally feels better than he has for several years.

Aside from the unusual features of the case in its anatomic phase it is remarkable in that all the symptoms were those of a chronic appendicitis with eventual perforation of the appendix and resulting peritonitis. The man's every symptom was typic of the appendical picture, and the case accentuates the belief that there are probably many cases of supposed appendical trouble resulting fatally under prolonged medical treatment which are not appendical at all, but due to some anomaly of the intestinal tract.

466 Franklin Street.

---

RHEUMATIC DYSMENORRHEA.

| | | |
|---|---|---|
| ℞ | Am. hydrochlor.................................... | 2½ ozs. |
| | Tr. stramonii.................................... | ½ oz. |
| | Tr. cimicifugæ.................................... | 1 oz. |
| | Syr. glycyrrhizæ.................................... | 2 drms. |
| | Tongaline.................................... | 6 ozs. |

M. Sig.—Teaspoonful three times a day.

## Figures and Tables

**Figure f1:**